# Patient safety and quality improvement education: a cross-sectional study of medical students’ preferences and attitudes

**DOI:** 10.1186/1472-6920-13-16

**Published:** 2013-02-05

**Authors:** Claire L Teigland, Rachel C Blasiak, Lindsay A Wilson, Rachel E Hines, Karen L Meyerhoff, Anthony J Viera

**Affiliations:** 1 Public Health Leadership Program, Gillings School of Global Public Health; School of Medicine, University of North Carolina at Chapel Hill, Chapel Hill, NC, USA; 2 Department of Internal Medicine, University of North Carolina at Chapel Hill, Chapel Hill, NC, USA; 3 Department of Family Medicine, University of North Carolina at Chapel Hill, Chapel Hill, NC, USA

**Keywords:** Medical education, Quality improvement, Patient safety, Curriculum development

## Abstract

**Background:**

Recent educational initiatives by both the World Health Organization and the American Association of Medical Colleges have endorsed integrating teaching of patient safety and quality improvement (QI) to medical students. Curriculum development should take into account learners’ attitudes and preferences. We surveyed students to assess preferences and attitudes about QI and patient safety education.

**Methods:**

An electronic survey was developed through focus groups, literature review, and local expert opinion and distributed via email to all medical students at a single medical school in the spring of 2012.

**Results:**

A greater proportion of students reported previous exposure to patient safety than to quality improvement topics (79% vs. 47%). More than 80% of students thought patient safety was of the same or greater importance than basic science or clinical skills whereas quality improvement was rated as the same or more important by about 70% of students. Students rated real life examples of quality improvement projects and participation in these projects with actual patients as potentially the most helpful (mean scores 4.2/5 and 3.9/5 respectively). For learning about patient safety, real life examples of mistakes were again rated most highly (mean scores 4.5/5 for MD presented mistakes and 4.1/5 for patient presented mistakes). Students rated QI as very important to their future career regardless of intended specialty (mean score 4.5/5).

**Conclusions:**

Teaching of patient safety and quality improvement to medical students will be best received if it is integrated into clinical education rather than solely taught in pre-clinical lectures or through independent computer modules. Students recognize that these topics are important to their careers as future physicians regardless of intended specialty.

## Background

Medical errors are a significant cause of morbidity and mortality. For example, in the United States (US), medical errors are thought to be responsible for more than 90,000 deaths annually [1,2]. Quality improvement (QI) has thus been a focus in the US and internationally in the past decade [2]. Both the World Health Organization (WHO) [3] and the American Association of Medical Colleges (AAMC) [4,5] have endorsed increased teaching of patient safety and QI during medical school. Despite policy consensus, few medical schools have implemented curricula addressing these topics, and there is little evidence on the best methods to teach them [6].

The majority of literature only reports investigations of post-graduate teaching of these topics [7]. Within the sparse literature about undergraduate medical education, a recent systematic review of patient safety curricula in medical schools demonstrated that most teaching about patient safety occurred during the third year, varied in length from 4–30 hours, and was taught by clinicians, ethicists, and medical education experts [8]. The authors concluded that current teaching does not meet the goals established by the WHO and includes no controlled assessments of a standardized curriculum (though results of a pilot study of the WHO curriculum are pending). One study of medical students’ preferences for learning about patient safety demonstrated that three-quarters of students polled supported discussions of real life mistakes, and 70% supported internet-based learning. Blogging and role-playing were much less popular [9]. Other research demonstrates that much of student learning regarding patient safety is informal and occurs as students witness mistakes [10]. Some authors have proposed that witnessing the various ways in which mistakes are handled is an effective way to integrate patient safety training into the clinical years of medical school [11]. One UK school implemented a 5-hour module on patient safety that included lectures, videos, and role playing, and found a year later that knowledge and perceived control over safety had improved, but attitudes had not [12].

Much of the literature regarding teaching quality improvement to medical students is from curricula implemented at individual schools. One school demonstrated that student-led diabetes QI projects both improved the care patients received and taught students QI principles [13]. Another school integrated QI teaching throughout all four years with introductory material presented through lectures in years one and two, physician-guided QI projects with community preceptors during the internal medicine clerkship, and case studies in the fourth year [14]. In order to better inform the development of a curriculum at our medical school, we conducted a survey that collected information regarding students’ attitudes and learning preferences regarding patient safety and QI education. Specifically, we sought to examine students’ preferences on timing, setting, and pedagogical methods for teaching these topics.

## Methods

### Survey development

First, we conducted a literature search to guide a series of three focus groups with medical students and key informant interviews with local experts in multiple medical specialties. We then used our findings to develop a 34 question electronic survey that was distributed to all medical students at the University of North Carolina School of Medicine via email from April 20, 2012 to May 07, 2012. We collected information on where and when students felt they were learning about patient safety and quality improvement in the current curriculum, how and when students they would prefer to learn about patient safety and quality improvement. We also asked for opinions on the importance of quality improvement education. A full list of survey questions can be found in Additional file 1; results from the questions assessing student knowledge of patient safety and quality improvement topics will be reported in a separate article. The Office of Human Research Ethics of UNC exempted the study from review. Students consented electronically to participate in this research study. Students who completed the survey were eligible to be entered into a drawing to win an iPad.

Students were asked to compare the relative importance of patient safety and quality improvement knowledge to basic science and clinical knowledge using a scale of 1 to 5 where 1 indicated “less important” [than basic science or clinical knowledge], 3 indicated the “same importance”, and 5 indicated “more important”. Students were also asked to choose when they would most like to be taught about these topics and to rate various methods of teaching on a scale of 1 to 5 with 1 representing “not-helpful” and 5 representing “very helpful”. Students were asked to also rate the importance of quality improvement to different medical specialties. Students were not asked these questions regarding patient safety because we did not think that any student would feel that patient safety was unimportant. The importance of quality improvement to each specialty was rated on a scale of 1 to 5 with one being “not important” and 5 being “very important”. Finally students were asked to respond to the following statement: “Quality improvement is important to my future as a physician” on a scale of 1 to 5 where 1 indicated “strongly disagree” and 5 indicated “strongly agree”.

### Data analysis

We report student characteristics, previous exposure to patient safety and quality improvement, preferences for curriculum timing and teaching method, and relative importance of patient safety and quality improvement in the medical school curriculum using frequencies or means with a standard deviation when appropriate. All analyses were completed in Stata 12 (StataCorp, College Station, Tx).

## Results

A total of 450 of 790 students participated in the survey, for a response rate of 57%. Overall, the demographics of students who participated in our survey were very similar to the medical student population in general. Seven percent of participants were black and 46% were male (Table 1), compared to 8% black and 49% male students in the entire medical school. Thirty-nine percent of participants were in their preclinical years (first or second year), 47% were in their clinical years (third or fourth year), and 14% were classified as other (research year, MPH or PhD years, or leave of absence). In the entire medical school, 42% were in their preclinical years, 44% were in their clinical years, and 13% were classified as other. Among participants, thirty-three percent of students were currently working on or already had an advanced degree, including an MPH, PhD, MBA, or MS. An MPH was the most commonly pursued advanced degree (42% of advanced degree students). Thirty-seven percent of students planned to go into primary care. Ten percent intended to enter a medical or pediatric specialty. Twenty-eight percent planned to go into general surgery or a surgical subspecialty. Twenty-six percent of students intended to enter other specialties (anesthesia, dermatology, emergency, medical psychology, neurology, nuclear medicine, pathology, physical medicine and rehabilitation, preventive medicine, psychology, radiology oncology, or radiology).

**Table 1:** Student characteristics (N = 352-358)

**Characteristic**	**n**	**Percent**
Race		
White	250	71%
Black	26	7.4%
Asian	48	14%
Hispanic	22	6.3%
Other	6	1.7%
Male	236	46%
Year in Medical School		
Preclinical	140	39%
Clinical	168	47%
Other	50	14%
Advanced Degree*	118	33%
Intended Specialty		
Intended primary care**	131	37%
Medical or pediatric subspecialty	35	10%
Surgical specialty***	98	28%
Other****	91	26%

*Currently working on or already has an advanced degree.

**Primary care specialties include: medicine, family medicine, medicine/pediatrics, and pediatrics.

***Surgical specialties included: general surgery, orthopedics, ophthalmology, otolaryngology, plastic surgery, thoracic surgery, vascular surgery, urology, obstetrics and gynecology, and neurosurgery.

****Other includes anesthesia, dermatology, emergency medicine, medicine-psychiatry, neurology, nuclear medicine, pathology, physical medicine and rehabilitation, preventive medicine, psychiatry, radiation oncology, and radiology.

Students’ exposure to and perceived importance of patient safety and quality improvement are reported in Table 2. More than three-quarters of students reported previous formal or informal exposure to patient safety, whereas only 47% of students reported previous exposure to quality improvement.

**Table 2:** Exposure to and importance of patient safety and quality improvement N = 369-450

**Question**	**Percent or Mean (SD)**
Previous exposure to Patient safety	79%
Previous exposure to Quality Improvement	47%
Relative importance of patient safety compared to basic science knowledge	57% More important
	34% Same importance
	9% Less important
	3.7 (SD 0.90)
Relative importance of patient safety compared to clinical knowledge	25% More important
	59% Same importance
	16% Less important
	3.1 (SD 0.79)
Relative importance of quality improvement compared to basic science knowledge	33% More important
	45% Same importance
	22% Less important
	3.2 (SD 0.88)
Relative importance of quality improvement compared to clinical knowledge	11% More important
	52% Same importance
	37% Less important
	2.7 (SD 0.76)

When comparing the importance of patient safety knowledge to basic science knowledge, the mean of students’ ratings was 3.7 out of 5 (SD 0.90). When comparing the importance of patient safety knowledge to clinical knowledge, the mean of students’ ratings was 3.1 (SD 0.79). When comparing quality improvement knowledge to basic science knowledge, the mean importance rating was 3.2 (SD 0.88). When comparing quality improvement knowledge to clinical knowledge, the mean importance rating was 2.7 (SD 0.76).

Student preferences for the timing of this education are reported in Table 3 for patient safety and Table 4 for quality improvement. Preferences for all students are reported as well as preferences stratified by pre-clinical and clinical students. Forty-seven percent of students preferred for patient safety education to be taught during clinical rotations, and twenty-seven percent of students preferred to be taught during the clinical skills class during first and second year. Students also preferred teaching about quality improvement to occur during the clinical years with more than 50% of those surveyed choosing this time period. 

**Table 3:** Student preference for patient safety education timing

**Teaching venue**	**All students N = 358**	**Pre-clinical N = 158**	**Clinical N = 200**
Pre-clinical lectures	12.0%	16.5%	8.5%
Community week*	6.4%	8.9%	4.5%
Medical humanities course	2.2	1.9%	2.5%
Clinical skill course	26.8	37.3%	18.5%
Clinical Rotations	46.9	27.9%	62%
Other	5.6	7.6%	4.0%

*Community week is an experience where 1^st^ and 2^nd^ year students go and spend a total of 5 weeks working one-on-one with a primary care preceptor somewhere in North Carolina.

**Table 4:** Student preference for quality improvement education timing

**Teaching venue**	**All students N = 357**	**Preclinical N = 158**	**Clinical N = 199**
Pre-clinical lectures	11.5	12.7	10.6
Community week*	7.6	11.4	4.5
Medical humanities course	4.8	7.0	3.0
Clinical skill course	17.9	24.7	12.6
Clinical Rotations	54.3	39.2	66.3
Other	3.9	5.1	3.0

*Community week is an experience where 1^st^ and 2^nd^ year students go and spend a total of 5 weeks working one-on-one with a primary care preceptor somewhere in North Carolina.

Students rated independent study including reading and reflection about patient safety topics as least helpful (2.2 out of a maximum of 5). Independently completed computer modules were also rated low with a mean of 2.4, followed closely by a large group lecture (2.6). The two most helpful methods of learning about patient safety were real-life examples of mistakes presented by a physician (4.5) or by patients (4.1) (Table 5). 

**Table 5:** Student preference for method of patient safety education*

**Teaching method**	**All students N = 383**	**Preclinical N = 158**	**Clinical N = 200**
Large Lecture	2.6 (1.1)	2.5 (1.1)	2.6 (1.1)
Real life example of mistakes and errors presented by physicians	4.5 (0.7)	4.4 (0.7)	4.5 (0.7)
Independently completed computer modules	2.4 (1.1)	2.1 (1.1)	2.5 (1.0)
Disclosing a medical error to a standardized patient	3.5 (1.1)	3.6 (1.0)	3.3 (1.1)
Real life examples of mistakes presented by patients	4.1 (1.0)	4.2 (1.0)	4.1 (1.0)
Independent study with reading and reflection	2.2 (1.1)	1.9 (1.1)	2.4 (1.1)
Problem based learning	3.2 (1.1)	3.2 (1.1)	3.3 (1.1)

* Preferences are rated on a scale of 1–5 with 1 being not helpful and 5 being very helpful.

Mean (SD).

Students were also asked to rate methods of quality improvement teaching on the same scale of not helpful (1) to very helpful (5) (Table 6). As seen with patient safety teaching, the methods rated least helpful were independent study with reading and reflection (2.3), independent computer modules (2.4), and large group lectures (2.6). The highest rated methods were physician-guided quality improvement projects on a real panel of patients (3.9) and real-life examples of quality improvement projects presented by physicians (4.2). 

**Table 6:** Student preference for method of quality improvement education*

**Teaching method**	**All students N = 366**	**Preclinical N = 158**	**Clinical N = 200**
Large Lecture	2.6 (1.2)	2.5 (1.2)	2.7 (1.2)
Real life example of quality improvement projects presented by physicians	4.2 (0.9)	4.1 (0.9)	4.2 (0.9)
Independently completed computer modules	2.4 (1.1)	2.1 (1.1)	2.5 (1.1)
Quality improvement project on fake patients	3.3 (1.1)	3.3 (1.1)	3.3 (1.1)
Quality improvement project on real patients	3.9 (1.0)	3.9 (1.0)	4.0 (1.0)
Independent study with reading and reflection	2.3 (1.1)	2.1 (1.1)	2.5 (1.1)
Problem based learning	3.2 (1.1)	3.1 (1.2)	3.3 (1.0)
Virtual Simulation (like the game Sim City)	3.3 (1.2)	3.3 (1.2)	3.3 (1.3)

* Preferences are rated on a scale of 1–5 with 1 being not helpful and 5 being very helpful.

Mean (SD).

Finally, students were asked to rate the importance of quality improvement to different medical specialties (Table 7). Perceived importance ratings of QI to all specialties listed were between 4.2 and 4.7, except for dermatology, which received an importance rating of 3.9 (Table 7). There was no significant difference between pre-clinical and clinical students’ opinions. Finally, when students rated the importance of quality improvement to their future career the average score was 4.5. Differences were examined between pre-clinical vs. clinical student categories, and by intended specialty; none of these comparisons proved to be significantly different. 

**Table 7:** Student rated importance of quality improvement to different medical specialties* mean (SD) N = 357

	**All students N = 357**	**Preclinical N = 157**	**Clinical N = 200**
Surgery	4.7	4.7	4.6
Pediatrics	4.5	4.5	4.4
Dermatology	3.9	3.9	3.8
Internal Medicine	4.5	4.5	4.5
Radiology	4.2	4.3	4.2
Obstetrics and Gynecology	4.6	4.6	4.6
Anesthesia	4.6	4.6	4.6
Emergency Medicine	4.6	4.6	4.6
Personal Career**	4.5	4.5	4.5

* Importance to each specialty was rated on a scale of 1–5 where 1 was not important and 5 was very important.

** Response to the following question: “Quality improvement is important to my future as a physician” on a scale of 1–5 were 1 was strongly disagree and 5 was strongly agree.

## Discussion

Within the last decade there has been a significant push to teach medical trainees about patient safety and quality improvement, in part prompted by landmark publications by the IOM [1,2]. While there has been an accumulating body of research regarding post-graduate education in these areas, less has been published regarding medical student education [7]. Our survey revealed that more than a decade after the IOM reports most medical students feel they are exposed to patient safety in school, but less than half reported having been exposed to quality improvement. Students do, however, recognize the importance of these topics, despite their varied exposure. In general, most students prefer to be taught these topics in clinical settings and using the most “hands-on” approaches possible. Lectures and independent study methods were very unpopular for learning both topics, while discussions of “real life” mistakes or participation in QI projects on real patients were rated as most helpful.

Student attitudes and learning preferences are important to consider when implementing a major curriculum change. An understanding of student attitudes helps educators predict whether a curriculum is liable to have “buy-in” or face resistance. If resistance is anticipated, education about the importance of the topic may be needed [15]. An understanding of learning preferences, although not the only factor to consider, can assist in prioritizing which pedagogical methods are most likely to be effective [16,17].

Interestingly, in contrast to the results by Thain and colleagues, who found that 70% of students preferred internet-based learning for patient safety, our students did not believe that computer modules would be helpful [9]. Our results are similar to Thain and colleagues in that students rated discussions of real life mistakes as most helpful. Likewise, students preferred quality improvement education to be more applied than simulated, preferring involvement in real QI projects to virtual simulation, independent study, or work on a “fake cohort” of patients. Perhaps the divergence in students’ attitudes towards internet-based learning is cultural as the studies were conducted in Singapore and the USA, respectively.

It seems clear that to engage medical students in these important topics, they should be delivered in as real an environment as possible. As some have suggested, the slow integration of these topics into medical curricula may be due to the lack of available educators or high cost [18]. Thus, involving medical students in “hands-on” QI projects may be cost-prohibitive, especially when medical school classes are large. However, a few medical schools have managed to involve medical students through introductory lectures early in medical school followed by participation in physician-guided QI projects during their third and fourth years. The results of these curriculum designs appear promising [13,14].

Our survey demonstrated broad student support for student participation in ongoing quality improvement projects. Additionally, research has shown that involving medical students in QI projects can not only aid student learning, but can also improve the quality of care for patients [13]. Thus integrating these projects into medical school curricula may be an effective use of resources on multiple levels.

Teaching obviously requires teachers with appropriate knowledge and skills in the topic. Therefore, faculty development is a crucial step before QI and patient safety curricula can be widely implemented. Given that post-graduate education has been quicker to integrate these topics into their education, perhaps soon there will be a new pool of faculty members armed with the knowledge necessary to teach medical students patient safety and quality improvement. Patient safety education may be aided by the help of health care administrators who have experience in the topic as one school in China demonstrated [19].

A feasible first step in patient safety education, favored by our students, and validated as an effective method by at least two schools, is the use of standardized patients to learn the art of error disclosure [20,21]. Medical students work with Standardized Patients (SPs) in the United States for the USMLE Step 2 CS, and most medical schools already have SPs integrated into portions of their curricula. Patient safety learning can also utilize the clinical years to turn the informal witnessing of mistakes into more formalized debriefing sessions and discussions about conflicting ways of dealing with errors as they occur [11].

Our study is limited by the potential for non-response bias. Students who answered this survey may have been more likely to be interested in patient safety and quality improvement. This greater interest may have led to inflation in the rating of attitudes and “helpfulness” ratings of the various teaching methods. However, responders were demographically similar to non-responders, which gives us some reassurance that non-response bias is likely to have had minimal effect. Additionally, more than half of the entire student body responded to the survey. Still, as this survey only represents the opinions of students from one US medical school, their preferences may not be generalizable to other schools.

## Conclusions

Overall our survey demonstrated that students are in favor of a new curriculum that formally addresses patient safety and quality improvement. Additional comments at the end of the survey left by students were overwhelmingly supportive. One comment in particular captures the essence of the overall student opinion: “Any situation that causes us to act, rather than simply read from papers or listen to lectures is going to be helpful. I learn by doing.” Medical schools should work to integrate these topics into clinical experiences rather than making them separate experiences in order to emphasize the importance of safety and quality to one’s career as a physician.

## Competing interests

None of the authors of this work have any financial or intellectual competing interests that would interfere with their interpretation of our results.

## Authors’ contributions

CLT participated in running and planning focus groups, performed key informant interviews, data analysis, survey design and distribution, and manuscript composition and revision. RCB: Participated in the literature review, running and planning focus groups, performed key informant interviews, survey question development, data analysis, and manuscript composition. LAW: Participated in running of focus groups, survey development, and IRB application. REH: RH participated in running and planning of focus groups, performed key informant interviews and data analysis. KLM: Participated in the literature review, running focus groups, helped with the IRB application, and contributed to data analysis. AJV: Conceived of the study, participated in survey design, supervised all aspects of the project including data analysis and IRB submission, provided critical revision of the manuscript, and provided funding for the survey drawing. All authors read and approved of the final manuscript.

## Pre-publication history

The pre-publication history for this paper can be accessed here:

http://www.biomedcentral.com/1472-6920/13/16/prepub

## Supplementary Material

Additional file 1 Patient safety and quality improvement curriculum development survey. Click here for file

## References

[B1] Kohn LT Corrigan J Donaldson MS To err is human: building a safer health system Natl Academy Pr 2000 6 1 312 25077248

[B2] America IoMCoQoHCi Crossing the quality chasm: A new health system for the 21st century 2001 National Academies Press 1 360 25057539

[B3] Walton M Woodward H Van Staalduinen S Lemer C Greaves F Noble D Ellis B Donaldson L Barraclough B The WHO patient safety curriculum guide for medical schools Qual Saf Health Care 2010 19 6 542 546 10.1136/qshc.2009.036970 21127112

[B4] Foundation NPS, Institute LL Unmet needs: teaching physicians to provide safe patient care 2010 National Patient Safety Foundation Accessed July 4th 2012. http://www.npsf.org/about-us/lucian-leape-institute-at-npsf/lli-reports-and-statements/the-patient-safety-imperative-for-health-care-reform/unmet-needs/

[B5] Batalden P Report V: contemporary issues in medicine: quality of care 2001 Washington, DC: Association of American Medical Colleges

[B6] Windish DM Reed DA Boonyasai RT Chakraborti C Bass EB Methodological rigor of quality improvement curricula for physician trainees: a systematic review and recommendations for change Acad Med 2009 84 12 1677 1692 10.1097/ACM.0b013e3181bfa080 19940573

[B7] Wong BM Etchells EE Kuper A Levinson W Shojania KG Teaching quality improvement and patient safety to trainees: a systematic review Acad Med 2010 85 9 1425 1439 10.1097/ACM.0b013e3181e2d0c6 20543652

[B8] Nie Y Li L Duan Y Chen P Barraclough BH Zhang M Li J Patient safety education for undergraduate medical students: a systematic review BMC Med Educ 2011 11 33 10.1186/1472-6920-11-33 21669007 PMC3128569

[B9] Thain S Ang SB Ti LK Medical students' preferred style of learning patient safety BMJ quality & safety 2011 20 2 201 10.1136/bmjqs.2009.039198 21303775

[B10] Fischer MA Mazor KM Baril J Alper E DeMarco D Pugnaire M Learning from mistakes. Factors that influence how students and residents learn from medical errors J Gen Intern Med 2006 21 5 419 423 10.1111/j.1525-1497.2006.00420.x 16704381 PMC1484785

[B11] de Feijter JM de Grave WS Dornan T Koopmans RP Scherpbier AJ Students' perceptions of patient safety during the transition from undergraduate to postgraduate training: an activity theory analysis Adv Health Sci Educ Theory Pract 2011 16 3 347 358 10.1007/s10459-010-9266-z 21132361 PMC3139877

[B12] Patey R Flin R Cuthbertson BH MacDonald L Mearns K Cleland J Williams D Patient safety: helping medical students understand error in healthcare Qual Saf Health Care 2007 16 4 256 259 10.1136/qshc.2006.021014 17693671 PMC2464940

[B13] Gould BE Grey MR Huntington CG Gruman C Rosen JH Storey E Abrahamson L Conaty AM Curry L Ferreira M Improving patient care outcomes by teaching quality improvement to medical students in community-based practices Acad Med 2002 77 10 1011 10.1097/00001888-200210000-00014 12377677

[B14] Ogrinc G Nierenberg DW Batalden PB Building Experiential Learning About Quality Improvement Into A Medical School Curriculum: The Dartmouth Experience Health Aff 2011 30 4 716 722 10.1377/hlthaff.2011.0072 21471493

[B15] Kotter JP Schlesinger LA Choosing strategies for change Harv Bus Rev 2008 86 7/8 130 10240501

[B16] McNulty JA Sonntag B Sinacore JM Evaluation of computer-aided instruction in a gross anatomy course: a six-year study Anat Sci Educ 2009 2 1 2 8 10.1002/ase.66 19217066

[B17] Phillips AS Settoon RP Phillips CR Enhancing a curriculum: A focus on the development process Coll Stud J 2008 42 4 1070 1074

[B18] DesHarnais SI Nash DB Reforming way medical students and physicians are taught about quality and safety Mt Sinai J Med 2011 78 6 834 841 10.1002/msj.20302 22069207

[B19] Leung GK Patil NG Ip MS Introducing patient safety to undergraduate medical students–a pilot program delivered by health care administrators Medical teacher 2010 32 12 e547 e551 10.3109/0142159X.2010.528810 21090942

[B20] Halbach JL Sullivan LL Teaching medical students about medical errors and patient safety: evaluation of a required curriculum Acad Med 2005 80 6 600 606 10.1097/00001888-200506000-00016 15917366

[B21] Gunderson AJ Smith KM Mayer DB McDonald T Centomani N Teaching medical students the art of medical error full disclosure: evaluation of a new curriculum Teach Learn Med 2009 21 3 229 232 10.1080/10401330903018526 20183343

