# Supplementary material for: Patient safety and quality improvement education: a cross-sectional study of medical students’ preferences and attitudes
Source: BMC Med Educ. 2013 Feb 5;13:16. doi: 10.1186/1472-6920-13-16 (PMC3570358; doi:10.1186/1472-6920-13-16)
Supplement: Additional file 1 — Patient safety and quality improvement curriculum development survey. [file 1472-6920-13-16-S1.pdf]

Additional File 1:

Patient Safety and Quality Improvement Curriculum Development Survey

This is a survey to help guide the development of UNC's medical student curriculum on patient safety and quality improvement.

This first set of questions is about patient safety.
Patient Safety is defined as: "Freedom from accidental or preventable injuries produced by medical care."

1. As compared to other medical students at my level of training I would rate my current knowledge of patient safety as

- ❍ 1 Poor
- ❍ 2
- ❍ 3 Average
- ❍ 4
- ❍ 5 Excellent

2. Compared with the importance of basic science knowledge, knowledge about patient safety is

- ❍ 1 less important
- ❍ 2
- ❍ 3 same importance
- ❍ 4
- ❍ 5 more important

3. Compared with the importance of clinical knowledge, knowledge about patient safety is

- ❍ 1 less important
- ❍ 2
- ❍ 3 same importance
- ❍ 4
- ❍ 5 more important

4. At any point during your medical curriculum, have you received formal education (e.g., lectures, small group teaching) about patient safety?

- ❍ Yes
- ❍ No
- ❍ Unsure

5.At any point during your medical training, have you received informal education (e.g., witnessing time-out before central line placement, observing proper hand-washing after exposure to patients with c. diff diarrhea) about patient safety?

- ❍ Yes
- ❍ No
- ❍ Unsure

6.In what setting did you learn about patient safety? Please check all that apply.

- ❑ Pre-clinical (MS1 and MS2) lectures
- ❑ Community Week
- ❑ Medicine and Society
- ❑ CSD/CSI or ICM
- ❑ Clinical Rotations (MS3 or MS4)
- ❑ Other (please specify) ___________________

7. At what point in medical school would you prefer to learn about patient safety?

- ❍ Pre-clinical (MS1 and MS2) lectures
- ❍ Community Week
- ❍ Medicine and Society
- ❍ CSD/CSI or ICM
- ❍ Clinical Rotations
- ❍ Other (please specify) ___________________

8. The following methods may be used to teach medical students about patient safety. Please indicate the degree to which you think each method would be helpful in teaching you about patient safety.

	Not Helpful 1	2	3	4	Very Helpful 5
Large lecture introducing patient safety	❍	❍	❍	❍	❍
Real life-examples of mistakes and medical errors presented by physicians	❍	❍	❍	❍	❍
Computer modules that introduce patient safety completed independently by students	❍	❍	❍	❍	❍
Standardized patient case in which you are required to disclose a medical error	❍	❍	❍	❍	❍
Real-life examples of mistakes presented by patients	❍	❍	❍	❍	❍
Independent study on patient safety that includes readings and reflection	❍	❍	❍	❍	❍
Problem-based	❍	❍	❍	❍	❍

learning in which a small group works through a patient-safety case					

We are interested in assessing students' current knowledge of Patient Safety. Please answer the following questions to the best of your ability

9. Most preventable errors in medical care are caused by:
- ❍ Knowledge deficiencies
- ❍ Process deficiencies
- ❍ Defensive practices

10. Approximately how many how many Americans die in hospitals each year due to mistakes in their care?
- ❍ Between 1,000 and 6,400
- ❍ Between 7,000 and 20,000
- ❍ Between 22,000 and 43,000
- ❍ Between 44,000 and 98,000
- ❍ Between 100,000 and 112,000

11. Two different paralytic agents, one with a long half-life and the other with a short half-life, are packaged in similar glass vials with blue caps. This is an example of:
- ❍ A forcing function
- ❍ A latent error
- ❍ A medication error
- ❍ Description error

12. Which one of the following is the best example of an active failure?
- ❍ Different blood pressure medications with similar bottles and labeling.
- ❍ An infusion pump that requires complex dosage calculations.
- ❍ Scheduling residents to work more than 60 hours in a row to cover a "power weekend."
- ❍ Overlooking a pneumothorax on a post-placement central line chest film.

13. Next, we'd like to ask you some questions about quality improvement. Quality Improvement is defined as: Efforts to make patient care safer, more effective, patient centered, timely, efficient, and equitable.

14. As compared to other medical students at my level of training I would rate my current knowledge of quality improvement as

- ❍ 1 poor
- ❍ 2
- ❍ 3 average
- ❍ 4
- ❍ 5 excellent

15. Compared with the importance of basic science knowledge, knowledge about quality improvement is

- ❍ 1 less important
- ❍ 2
- ❍ 3 same importance
- ❍ 4
- ❍ 5 more important

16. Compared with the importance of clinical knowledge, knowledge about quality improvement is

- ❍ 1 less important
- ❍ 2
- ❍ 3 same importance
- ❍ 4
- ❍ 5 more important

17. At any point during your medical curriculum, have you received formal education (e.g., lectures, small group teaching) about quality improvement?

- ❍ Yes
- ❍ No
- ❍ Unsure

18. At any point during your medical training, have you received informal education (e.g., observe part of a PDSA cycle during community week, hear residents discuss their quality improvement projects) about quality improvement?

- ❍ Yes
- ❍ No
- ❍ Unsure

19. In what setting did you learn about quality improvement? Please check all that apply.

- ❍ Pre-clinical (MS1 and MS2) lectures
- ❍ Community Week
- ❍ Medicine and Society
- ❍ CSD/CSI or ICM
- ❍ Clinical Rotations (MS3 and MS4)
- ❍ Other (please specify) ____________________

20. At what point in medical school would you prefer to learn about quality improvement?

- ❍ Pre-clinical (MS1 and MS2) lectures
- ❍ Community Week
- ❍ Medicine and Society
- ❍ CSD/CSI or ICM
- ❍ Clinical Rotations (MS3 and MS4)
- ❍ Other (please specify) ____________________

21. The following methods are used to teach medical students about quality improvement. Please indicate the degree to which you think each method would be helpful in teaching you about quality improvement.

	Not Helpful 1	2	3	4	Very Helpful 5
Large lecture introducing quality improvement	❍	❍	❍	❍	❍
Real life-examples of quality improvement projects presented by physicians	❍	❍	❍	❍	❍
Computer modules that introduce quality improvement completed independently by students	❍	❍	❍	❍	❍
Faculty-guided quality improvement project on a fake cohort of patients	❍	❍	❍	❍	❍
Faculty-guided quality improvement project with a cohort of real patients	❍	❍	❍	❍	❍
Independent study on patient QI that includes readings and reflection	❍	❍	❍	❍	❍

Problem-based learning in which a small group works through a quality-improvement case	❍	❍	❍	❍	❍
Virtual simulation (e.g., like the game Sim City)	❍	❍	❍	❍	❍

22. Please rate the importance of quality improvement in the following medical specialties.

	Not Important 1	2	3	4	Very Important 5
Surgery	❍	❍	❍	❍	❍
Pediatrics	❍	❍	❍	❍	❍
Dermatology	❍	❍	❍	❍	❍
Internal Medicine	❍	❍	❍	❍	❍
Radiology	❍	❍	❍	❍	❍
OB GYN	❍	❍	❍	❍	❍
Anesthesia	❍	❍	❍	❍	❍
Emergency Medicine	❍	❍	❍	❍	❍

23.   Please indicate whether your level of agreement to the following statement. Quality improvement is important to my future as a physician.

- Strongly Disagree
- Disagree
- Neither Agree nor Disagree
- Agree
- Strongly Agree

We are interested in assessing students' current knowledge of Quality Improvement. Please answer the following questions to the best of your ability.

24. Where does the United States rank among 19 industrialized nations, when it comes to deaths from preventable and treatable conditions (with first being lowest and last being the highest rate of deaths)?

- First
- Fifth
- Ninth
- Nineteenth

25. Which of the following improvement efforts is the best example of increasing the effectiveness of care?

- Decreasing adverse drug events by having a pharmacist on rounds.
- Shortening wait times at a clinic by allowing patients to self-register on a computer in the waiting room
- Improving the percent of clinic patients achieving their goal blood pressure by instituting a series of reminders for providers about evidence-based processes
- Instituting quarterly focus groups of patients seen in the emergency department to better identify patient concerns
- Equalizing rates of cardiac workups between men and women presenting to the Emergency Department with chest pain through staff development and weekly feedback about workup rates

26. Which of the following improvement efforts is the best example of increasing the equity of care?

- ❍ Decreasing adverse drug events by having a pharmacist on rounds.
- ❍ Shortening wait times at a clinic by allowing patients to self-register on a computer in the waiting room
- ❍ Improving the percent of clinic patients achieving their goal blood pressure by instituting a series of reminders for providers about evidence-based processes
- ❍ Instituting quarterly focus groups of patients seen in the emergency department to better identify patient concerns
- ❍ Equalizing rates of cardiac workups between men and women presenting to the Emergency Department with chest pain through staff development and weekly feedback about workup rates

27. Which of the following is a basic principle of improvement?

- ❍ Improvement must come from the bottom up—not the top down
- ❍ Every system is perfectly designed to get the results it gets.
- ❍ Data should drive improvement.
- ❍ When examining a complex system, consider all the parts independently.

28. Having a clear aim statement is important in quality improvement work because:

- ❍ Aim statements provide a clear and specific goal for the organization to reach.
- ❍ Grant agencies require clear aim statements when they are considering funding requests.
- ❍ Aim statements make the change process move along more rapidly.
- ❍ The leaders of most organizations expect to see these types of goals.

To help us better interpret our results, we would like to know a bit more about you. Thank you.

29. Race/ethnicity:

- ❍ White
- ❍ Black
- ❍ Asian
- ❍ Hispanic
- ❍ Other

30.Sex:

- ❍ Male
- ❍ Female

31. What year in medical school are you?

- ❍ First year
- ❍ Second year
- ❍ Third year
- ❍ Fourth year
- ❍ LOA
- ❍ MD/PhD student during PhD years

31. (a) What are you doing during your LOA year?

- ❍ Research
- ❍ MPH
- ❍ Other

32. Do you have an advanced degree, or are you currently working on an advanced degree?

- ❍ Yes
- ❍ No

If yes to 32:

32. (a) What type of advanced degree do you have or are you working on?

- ❍ PhD
- ❍ MPH
- ❍ MBA or MHA
- ❍ JD
- ❍ Other (please specify) ___________________

If no to 32:
32(b) Do you intend to get an advanced degree?

- ❍ Yes
- ❍ No

If Yes to 32(b):
32 (b, i) What degree do you plan to obtain?

- ❍ MPH
- ❍ PhD
- ❍ MBA or MHA
- ❍ JD
- ❍ Other (please specify) ___________________

33. Have you ever been hospitalized?

- Yes
- No

34. What is your intended career path? If you are currently undecided, please choose which specialty is currently top on your list.

- Anesthesiology
- Dermatology
- Emergency Medicine
- Family Medicine
- General Surgery
- Internal Medicine
- Medical Subspecialty
- Med/Peds
- Med/Psych
- Neurological Surgery
- Neurology
- Nuclear Medicine
- OB/GYN
- Ophthalmology
- Orthopedic Surgery
- Otolaryngology
- Pathology
- Pediatrics
- Pediatric Subspecialty
- PM&R
- Plastic Surgery
- Preventive Medicine
- Psychiatry
- Radiation Oncology
- Radiology
- Thoracic Surgery
- Urology
- Vascular Surgery

If you have any ideas, suggestions, or comments about a patient safety and quality improvement curriculum in medical school, please add them in the space provided below:

Thanks for your participation. If you would like to be entered into the drawing for the iPad, please enter your email address below.
